# Application of a sensitive collection heuristic for very large protein families: Evolutionary relationship between adipose triglyceride lipase (ATGL) and classic mammalian lipases

**DOI:** 10.1186/1471-2105-7-164

**Published:** 2006-03-21

**Authors:** Georg Schneider, Georg Neuberger, Michael Wildpaner, Sun Tian, Igor Berezovsky, Frank Eisenhaber

**Affiliations:** 1 IMP - Research Institute of Molecular Pathology, Dr. Bohr-Gasse 7, A-1030 Vienna, Republic of Austria; 2 Department of Chemistry and Chemical Biology, Harvard University, 12 Oxford str., M-105, 02138 Cambridge, MA, USA

## Abstract

**Background:**

Manually finding subtle yet statistically significant links to distantly related homologues becomes practically impossible for very populated protein families due to the sheer number of similarity searches to be invoked and analyzed. The unclear evolutionary relationship between classical mammalian lipases and the recently discovered human adipose triglyceride lipase (ATGL; a patatin family member) is an exemplary case for such a problem.

**Results:**

We describe an unsupervised, sensitive sequence segment collection heuristic suitable for assembling very large protein families. It is based on fan-like expanding, iterative database searches. To prevent inclusion of unrelated hits, additional criteria are introduced: minimal alignment length and overlap with starting sequence segments, finding starting sequences in reciprocal searches, automated filtering for compositional bias and repetitive patterns. This heuristic was implemented as FAMILYSEARCHER in the ANNIE sequence analysis environment and applied to search for protein links between the classical lipase family and the patatin-like group.

**Conclusion:**

The FAMILYSEARCHER is an efficient tool for tracing distant evolutionary relationships involving large protein families. Although classical lipases and ATGL have no obvious sequence similarity and differ with regard to fold and catalytic mechanism, homology links detected with FAMILYSEARCHER show that they are evolutionarily related. The conserved sequence parts can be narrowed down to an ancestral core module consisting of three β-strands, one α-helix and a turn containing the typical nucleophilic serine. Moreover, this ancestral module also appears in numerous enzymes with various substrate specificities, but that critically rely on nucleophilic attack mechanisms.

## Background

The failure to develop a rational, generally applicable cure for obesity-related diseases can be attributed to the highly complex regulation of energy metabolism, which is not yet fully understood. On the other hand considering the historic successes in deciphering the underlying biochemical pathways, it is assumed that the chemical transformation steps of basic metabolites are known in their entirety. This view is seriously questioned in light of the recent discovery of ATGL, a protein that catalyzes the initial step of hydrolysis of triacylglycerides at the surface of lipid droplets in adipocytes [1]. It is surprising that the fundamental activity of this key enzyme escaped from attention so far [2,3]. Just considering the many dozens of additional hypothetical human protein sequences with low but statistically significant sequence-similarity to known metabolic enzymes that can be collected with PSI-BLAST searches [4], more such findings are still expected to be ahead.

One of the key steps in energy metabolism is the separation of fatty acids from glycerol moieties. A diverse set of lipases performs this task in various contexts by hydrolyzing the connecting ester-bonds [5]. One of the best characterized lipases, pancreatic lipase, acts at the stage of food digestion [6]. Other lipases, such as hormone sensitive lipase or lipoprotein lipase, are involved in lipid accumulation and release in tissue [7,8].

Most lipases share a common type of 3D structure known as α/β-hydrolase fold, which is present in enzymes with quite diverse substrate specificities [9,10]. The catalytic mechanism of most lipases is reminescent of serine proteases as it proceeds via the nucleophilic attack of a serine-histidine-aspartate triad [10].

The recently discovered, novel key enzyme involved in fatty acid release from adipocytes, adipose triglyceride lipase (ATGL) [1], does not share any direct sequence similarity with known mammalian lipases. In fact, it appears to belong to a protein family that is centered around patatin, a potato storage protein with lipid acyl hydrolase activity [11,12]. The catalytic mechanism of these enzymes is inherently different from classic lipases as it proceeds via a serine-aspartate dyad [13,14] as opposed to the well described serine-histidine-aspartate triad.

In this work, we present sequence-analytic evidence that the ATGL/patatin family and the classic mammalian lipases represented by the human pancreatic lipase evolved from a common ancestor. Moreover, we display a set of structural and sequence key features that are conserved between these two enzyme groups including also related protein families.

The analysis of homology relationships within large superfamilies of protein sequences are a reoccurring theme in biomolecular sequence analysis. Finding the pancreatic lipase/ATGL relationship is just one application for the respective methodologies. It should be noted that detecting subtle yet statistically significant and structurally plausible relationships in families involving thousands of members is not a straightforward task since the manual analysis of myriads of reports generated by standard BLAST/PSI-BLAST [4] installations for sequence comparisons in databases is impossible in practice. Progress in this area was hampered by insufficiently developed tools. Here, we developed a computer implementation of a family searching heuristic involving: (i) Automated invocation of fan-like iterative PSI-BLAST [4] searches with starting sequences. (ii) Filtering of starting sequences with various sequence-analytic methods for detecting compositional and repetitive pattern bias. (iii) Automatic re-detection of starting sequence segments in reciprocal searches. (iv) Criteria for alignment length and overlap with the starting sequence segments. (v) Automated parsing of outputs and (vi) database-supported analysis of similarity networks. The user-parameterized measures (ii-iv) are designed to suppress the detection of unrelated hits for the case of a starting sequence that are thought to represent a single globular domain, a functionally and structurally independent elementary module. This FAMILYSEARCHER is part of the sequence-analytic workbench ANNIE [15] that is being developed in our laboratory. To our knowledge, this article describes the first software package for sequence family collection with fully automated checks for bidirectional search criteria, transitive hit overlap criteria and generic procedures for masking repetitive regions that is applicable for extremely large sequence families.

## Results

### FAMILYSEARCHER: Methodical specifics of analyzing homology relationships in large sequence families

The concept of sequence homology is a powerful approach to organize the sequence space of known proteins and to generate hypotheses on the function and evolutionary origin of yet uncharacterized proteins [16]. If a protein sequence segment "A" without compositional or pattern bias is used as a starting point in a search for similar proteins and reveals a sequence "B" as a hit, the respective two sequences are considered homologous and a common evolutionary ancestor can be postulated. The direct connection between these two sequences is called a unidirectional link if "A" finds "B" and a bidirectional one if the reciprocal search started with "B" also reveals "A" as homologue. If two protein sequence segments "A" and "B" do not have a direct link but are significantly similar to the same sequence region of an intermediary protein "I", all three sequences are considered homologous. This relationship can be generalized for the case of multiple intermediates. The sequence of links in the sequence space relating two homologous proteins is termed a path.

Studying distant homology relationships of densely populated protein clusters of the sequence space with thousands of similar sequences is a complicated endeavor. BLAST/PSI_BLAST searches [4] are not commutative with respect to exchanges of starting and hit sequences and slightly differing queries can collect largely different families. Therefore, only exhaustive searches involving all potential family members as start sequences can assure that the maximal number of sequence family members is found. This procedure also ensures that non-trivial paths to new proteins that are unexpectedly related are determined. For the analysis of large families, this implies thousands or tens of thousands of database searches. Although performance and costs of compute servers and storage devices have improved, it is still early to launch such calculations without well-thought selection of a starting protein segment (cluster-based compute-server costs of days/weeks and storage needs in 10^-2^-10^1 ^TB). Besides the hardware issues, software solutions are necessary to automatically guide the search process and to analyze the huge amount of data generated.

Within our ANNIE suite [15], we have developed FAMILYSEARCHER, a generic environment for protein sequence family collection (see Methods for details). The procedure is organized in stages. At the beginning of each stage "n", the starting sequence segments (at the first stage n = 1: the user-defined segment) are freed from regions with compositional and repetitive pattern bias and PSI-BLAST [4] searches are started with them. Hit segments are collected, parsed and the new hits represent the start sequences for the next stage "n+1". It is possible to exclude candidates that are not confirmed by reciprocal checks; i.e., the requirement that the starting sequence at stage "n+1" should find back the same region of the starting sequence at stage "n" that lead to its own selection as a hit (establishment of bi-directional hits). After completion of a predefined maximal number of stages (or the procedures converge prematurely if no new hits are found), the links are stored in a database and paths between hits are analyzed. This strategy has already been successfully applied to reconstruct entire sequence families for smaller sequence groups (for example [17-20]) in similar contexts where a simpler procedure was applied.

### Paths of links in the sequence space with statistically significant sequence similarity between the groups of ATGL/patatin and classical lipases do exist

**Figure 1:**
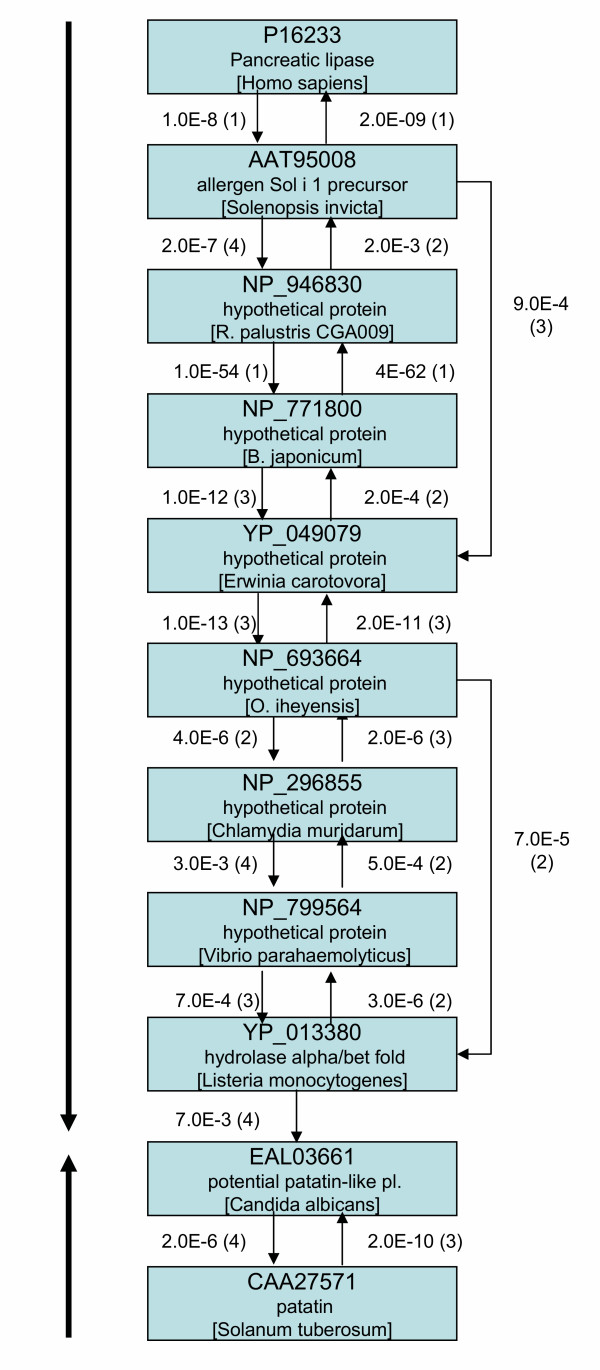
**Set of nine intermediate sequences linking pancreatic lipase with patatin**. The accessions of the two starting sequences of FAMILYSEARCHER and the nine intermediates are enclosed in boxes. The large arrows to the left side represent path components found by FAMILYSEARCHER using the respective global starting sequences. The thin arrows between boxes represent directional links found with PSI-BLAST [4] (direction from starting sequence to hit) and are labeled with the corresponding round number of first significant detection (in parentheses) and the inclusion E-value. In addition to the full path, two shortcuts are displayed. The respective alignment regions are (from the top to the bottom, segment boundaries in brackets): AAT95008 [79–249], NP_946830 [63–209], NP_771800 [64–211], YP_049079 [81–177], NP_693664 [158–398], NP_296855 [21–118], NP_799564 [77–124], YP_013380 [8–60] via direct path and [9–119] via shortcut, EAL03661 [423–518] from the YP_013380 side and [397–579] from patatin side. It is important to assure that the connection to EAL03661 and the other intermediates is in the same region regardless of the side of the path (pancreatic lipase or patatin) the search was started. Examining the corresponding PSI-BLAST generated alignments reveals that the boundaries of the alignment starting from the pancreatic side (423 – 518) are completely contained within the alignment starting from the patatin side (397 – 579) of the path.

To search for a connection between classic lipases and ATGL, we selected the lipolytic domains of one well-characterized protein for each sequence family as a starting sequence set. We used regions of the pancreatic lipase (accession: P16233, residues 17–351) and of the potato tuber protein patatin (accession: CAA27571, residues 32–320) for two separate familysearcher processes. After performing up to seven stages of the collection procedure for either of the FAMILYSEARCHER processes (i.e., iterative PSI-BLAST searches from both directions; in total 30598 individual searches and 22082 protein hits with paths consisting at least of unidirectional links to any of the two starting sequences), we indeed obtained a set of 12662 paths that connect these two query sequences. For one set of nine intermediate sequences establishing a connection between the two starting targets, several paths are depicted in Figure 1.

There are paths that consist fully of bidirectional links (i.e., the similarity relationship is confirmed by reciprocal searches) with the sole exception of the links connecting a representative of the classical lipase group to the potential patatin-like phospholipase EAL03661. For example (see Figure 1), the connection between YP_013380 (hydrolase, α/β fold) and EAL03661 (potential patatin-like phospholipase) can only be established from the pancreatic lipase side of the path. Closer examination of significances reveals that the links at this stage are also among the weakest and appear to represent the "missing link" between the two sequence families. There are other paths between the two starting sequences avoiding EAL03661 but the respective significances are worse and the alignments are shorter (data not shown). Known 3D structures that are most closely related to the proteins included in the path of Figure 1 are listed in Table 1. Obviously, the SCOP and CATH identifiers above EAL03661 correspond to variations within a single superfamily (c.69.1 and 3.40.50.1820 respectively). The step to the ATGL/patatin group involves a change in fold (SCOP c.19.1.3). Figure 1 also shows that there are several "shortcuts". These are links between non-adjacent intermediates in the path with a maximal fraction of bidirectional links that, although being significant, lack a confirmation from reciprocal searches. Known 3D structures that are most closely related to the proteins included in the path of Figure 1 are listed in Table 1.

It should be noted that the FAMILYSEARCHER run had to be halted because of computational time and storage space constraints. At the given point, the algorithm had been running on 70 CPUs for 10 days while it had produced about 1 Terabyte of data. Since the main aim of uncovering a link between the two families of lipases had been accomplished (a number of potential links had already been found), it was decided to stop the run and investigate the obtained results in detail. It can be expected that other "missing links" or even further related protein families remain to be found despite of the variety of proteins and enzymes that were already detected to be related to the two lipase families.

### The ancestral module common to the ATGL/patatin and classical lipase families consists of a three-strand β-sheet, an α-helix and a turn with the active site serine

**Figure 2:**
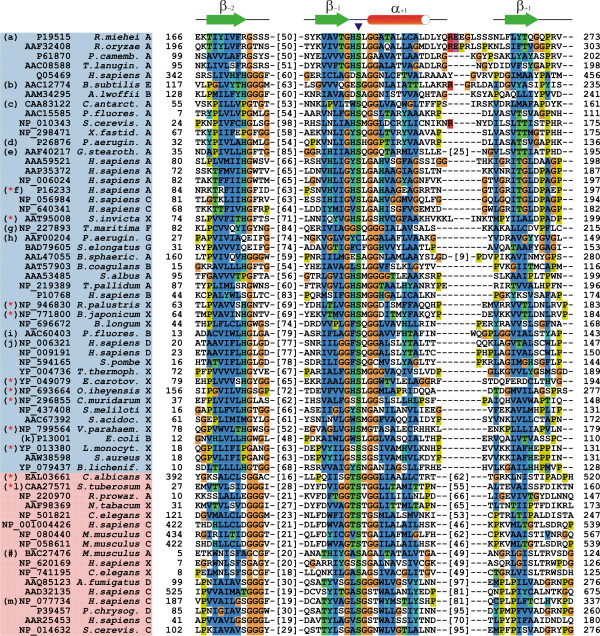
**The conserved ancestral core module: Multiple alignment**. The sequences are described to the left by their NR accession followed by the species name and an upper-case letter representing the annotated substrate specificity (A for lipases, B for esterases in general, C for phospholipases, D for lysophospholipases, E for a Brefeldin A esterase, F for acetyl xylan esterases, G for dienelactone hydrolases, X for unknown). The alignment includes the path sequences from figure 1 (marked by red asterisk between parentheses (*) in front of the accessions) in addition to proteins from most major clusters. Sequences with resolved crystal structures are shown with a lower-case letter between parentheses to the left. The corresponding PDB accessions are (a) 4TGL [22], (b) 1JKM [23], (c) 1TCB [24], (d) 1EX9 [25], (e) 1KU0 [26], (f) 1N8S [27], (g) 1VLQ (unpublished), (h) 1DIN [28], (i) 1AUO [29], (j) 1FJ2 [30], (k) 1M33 [31], (l) 1OXW [13] and (m) 1CJY [14]. The upper part of the sequence description with the blue background shows proteins from the classic α/β-hydrolase fold family, the lower part with the red background shows sequences from the ATGL/patatin group. The alignment to the right shows the secondary structural elements from the core module: the β-strands β_-2_, β_-1_, β_+1 _and the α-helix α_+1_. These helices and sheets are depicted above in their typical lengths as judged from the included 3D structures. Central part of the alignment is the nucleophilic elbow with the archetypical GXSXG sequence. The location of the nucleophilic residue (mainly serine, but two dienelactone hydrolases with active cysteines are also shown) is indicated by a blue triangle on top of the alignment. The numbers that flank the alignment show the start and end positions of the displayed subsequences with regard to the corresponding entries. The numbers in brackets between the secondary structural elements represent amino acids that are not shown in the alignment.

**Figure 3:**
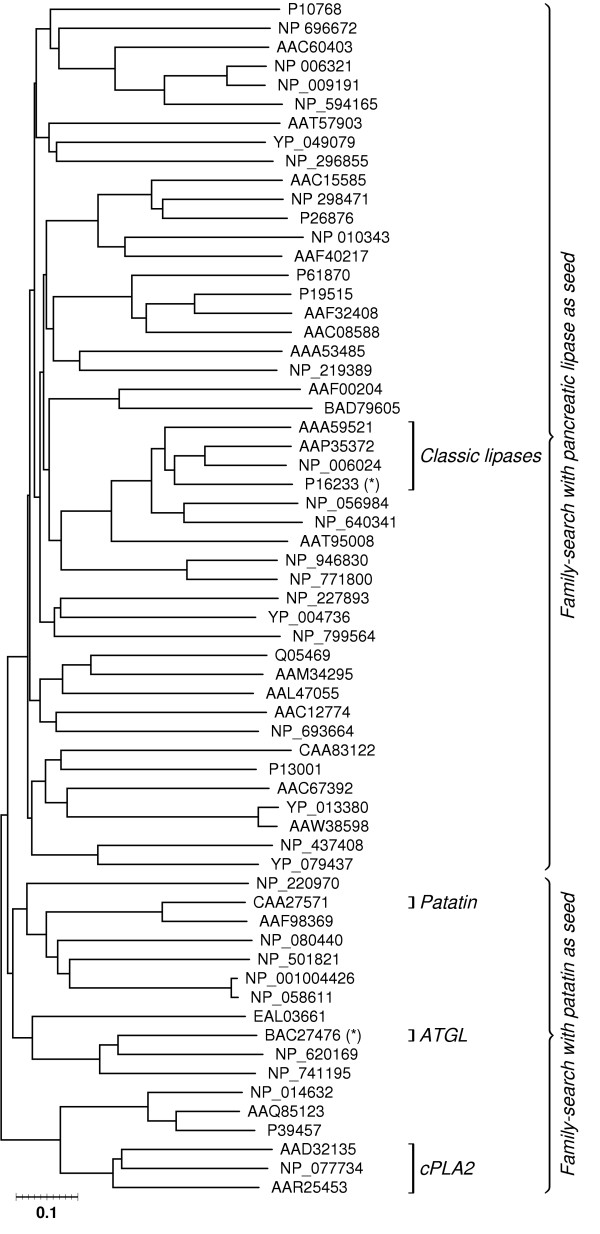
**The conserved ancestral core module: The phylogenetic tree**. The tree depicts the evolutionary relationship between the sequence segments that are included in the multiple alignment (Figure 2). The upper part of the tree contains the protein segments that were found with pancreatic lipase as seed for the FAMILYSEARCHER procedure, while the lower part contains the results of the patatin family-search. The starting sequences for the iterative procedures each have an asterix between parentheses (*) after the accession number. Important spots of the tree are marked by a bracket that is followed by a short description. Here, the classic lipases in the alignment are hepatic lipase (AAA59221), lipoprotein lipase (AAP35372), endothelial lipase (NP_006024) and pancreatic lipase (P16233). The bottom of the tree also contains three type A2 cytosolic phospholipases. Besides patatin (1OXW, [13]), this is the second type of protein from the pooled results that has been shown to function via a catalytic dyad (1CJY, [14]).

The set of protein segments collected by FAMILYSEARCHER has a common region of homology that forms the basis for the path in the sequence space connecting the pancreatic lipase group with patatin/ATGL. This common region can be distilled by analyzing the PSI-BLAST generated pairwise alignments, by 3D structural considerations and by investigating conservation patterns in sequence subfamilies (see Methods). An alignment of the respective sequence regions involving the path members from Figure 1 (11 sequences), representatives from most major clusters as well as their most similar sequences with atom-level resolved 3D structure is shown in Figure 2 (in total 63 sequences). The common region of similarity involves 50–70 residues and is sometimes interrupted by inserts. We suggest that these 50–70 residues represent an ancestral sequence module that, at the beginning of its evolution, might have been without inserted regions. It is interesting to note that this module contains only a part of the catalytic core, namely the nucleophilic serine, which is either involved in an enzymatic triad (proteins found with pancreatic lipase as seed) or dyad mechanism (proteins found with patatin as seed). The sequence regions that encompass the remaining catalytically active residues are too divergent among many subfamilies to deduce an ancestral relationship and, thus, could not be incorporated into the multiple alignment, which represents the whole sequence group (see also structural analyses of spatial location conservation further below). The phylogeny of the sequence segments from the alignment (Figure 3) is in agreement with the supposed evolutionary position of the intermediate sequences found in the path.

From the viewpoint of molecular function, these homologues are not limited to lipases and esterases, but include large sets of proteins that act on various other types of substrates. Among these enzymes are proteins such as polyketide synthases, dienelactone hydrolases or aminopeptidases (see legend to Figure 2 and data not shown). As we did not reconstruct the entire encompassing protein family, we expect that more proteins with alternative substrate specificities might be detected in rigorous searches. Nevertheless, the variety of enzymes found in this work clearly demonstrates the ubiquitousness of the ancestral module.

**Figure 4:**
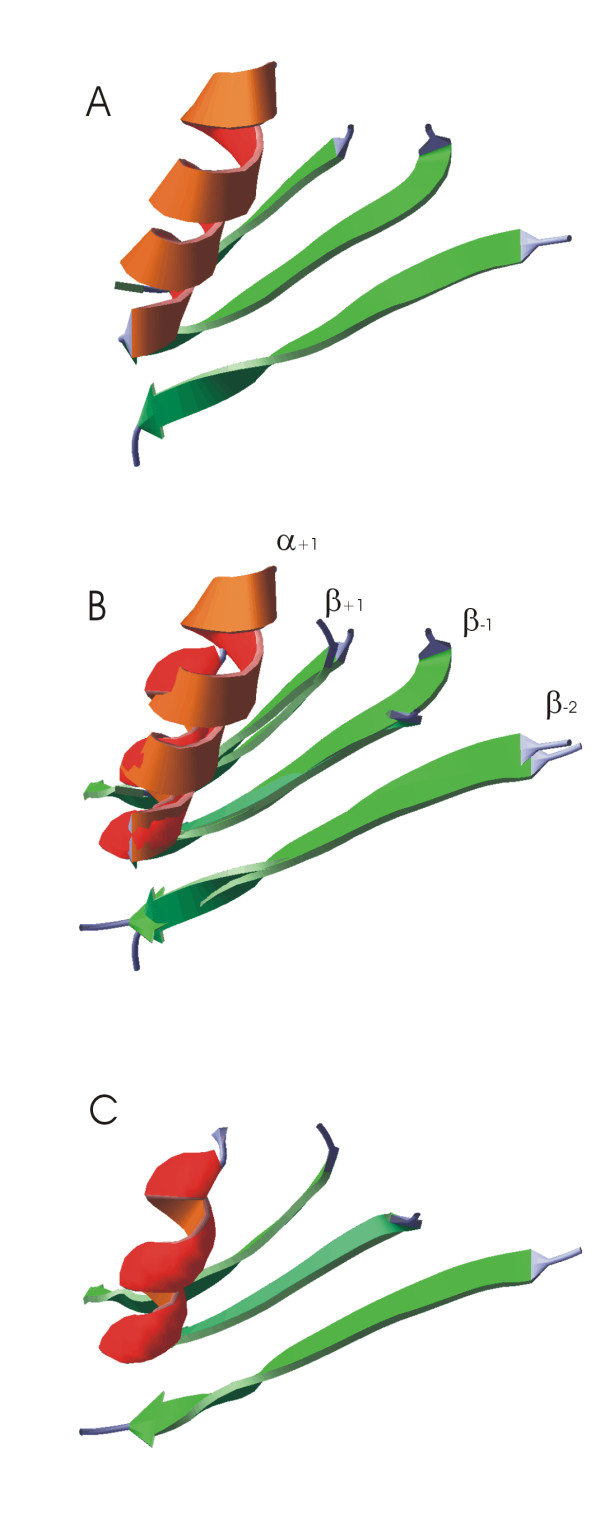
**Structural superposition of the ancestral core in pancreatic lipase and patatin**. 3D structures of the conserved core module (see Figure 2) from pancreatic lipase 1N8S [27] (A, segments 68–77, 144–166, 170–176), patatin 1OXW [13] (C, segments 28–37, 70–87, 148–158) and a superposition of both (B). The module starts with a β-strand (β_-2_) that is followed by another β-strand (β_-1_), an α-helix (α_+1_) and a β-strand (β_+1_). Numbers in parentheses indicate the relative positions to the nucleophilic elbow in the protein sequence. This loop is crucial for catalytic activity and typically contains a catalytic serine. It is located between the adjacent secondary structure elements β_-1 _and α_+1_. The connections to the other β-strands (β_-2 _and β_+1_) are not direct and can include longer stretches of additional secondary structure.

Visual inspection of 3D structures of proteins encountered during the search at regions involved in the alignment of Figure 2 reveals a set of conserved structural elements. These consist of three parallel β-strands and an α-helix located between the second and third strand. The characteristic nucleophilic elbow of esterases is located in the loop between the end of the second beta-strand and the start of the alpha-helix. Considering the locations of the secondary structural elements with respect to the nucleophilic elbow, we introduce the following numbering: β_-2 _and β_-1 _for the strands that are upstream of the nucleophilic residue, and α_+1 _and β_+1 _for the structures that lie downstream of the elbow. The core structural elements are depicted in Figure 4.

Only few automated structure comparison tools such as CE [21] can delineate common substructures from otherwise differing protein structures. It is interesting to note that this program does not find the ancestral core module if it is confronted with the complete 3D structures of the respective proteins. We generated pairwise structural superposition of a set of 13 3D structures (set of 11 structures – 4TGL [22], 1JKM [23], 1TCB [24], 1EX9 [25], 1KU0 [26], 1N8S [27], 1VLQ (unpublished), 1DIN [28], 1AUO [29], 1FJ2 [30], 1M33 [31] – from proteins obtained in the family-search using pancreatic lipase as the query, and a set of two structures – 1OXW [13], 1CJY [14] – from the ATGL-side). For the 55 superposed pairs of α/β-hydrolase structures belonging to the pancreatic lipase group, 32 aligned with scores at family level similarity (Z-score > 4.5), 11 with superfamily level similarity (Z-score between 4.0 and 4.5), 6 in the twilight zone (Z-score between 3.7 and 4.0), and 6 with low significance similarity with Z-scores between 2.6 and 3.7. Moreover, the secondary structural elements β_-1 _and α_+1 _including the active nucleophilic residue were correctly aligned for each pair of structures, regardless of the significance level. The same observation could be made for the 2 structures from the ATGL-side (high, family-level Z-score = 5.0). However, no alignment could be generated by CE between any structure from the classic lipase set and any from the ATGL side. We concluded that fold similarities are restricted to a small part of the structure – the conserved ancestral module – and that this stretch is simply too short to provide significant results because the remainder of the fold could not be aligned by the CE program.

To test this hypothesis, we generated structural alignments between the set of 11 structures and 2 different sub-stretches of the crystal structures from the ATGL-side: (i) The entire core module ranging from β_-2 _to β_+1 _including also inserted secondary structure elements (1CJY: Val187 – Lys335, 1OXW: Leu25 – Lys158). (ii) The part of the core module that encompasses the nucleophilic elbow from β_-1 _to α_+1 _(1CJY: Ala221 – Ser239, 1OXW: Phe70 – Ser87). In the former case, a nearly correct structural superposition (max. shift of 0–3 residues in the nucleophilic elbow) could be obtained for about half of the alignments, yet with low-significance similarity levels corresponding to Z-scores between 1.6 and 3.7. In the latter case, all core elements were correctly aligned to the subject structures. The low Z-scores of 2.6–2.8 obtained in this context are a result of the shortness of the stretch. As the significance measure is dependent on the length of the region, even the cores of the highly similar structures 1CJY and 1OXW align with a Z-score of only 3.1. These results indicate that structural similarities limited to the ancestral core module do exist, even though fold and sequences may be different for classic lipases and ATGL.

## Discussion and conclusion

The analyses of homology relationships between sequences of large superfamilies were previously hampered by the insufficiency of the available computer-based methods and corresponding tools. For example, confirming an evolutionary relationship between classical lipases clustered around pancreatic lipase and the ATGL/patatin group is a difficult task not only because the relationship is distant, the similarity is subtle and the respective common region involves a substructure interrupted with insertions. The group of sequences that are very similar to classical lipases is so large (with tens of thousands of members) that most database searches started with their representatives get obliterated with closely related group members. It becomes a major problem to identify proteins that are located at the boundary of this cluster and give hope to discover new links to outside protein groups if used as a starting sequence. On the other side, sequence diversity among the ATGL/patatin group is obviously not large enough to generate a profile that is sufficiently rich to establish the link to classical lipases. Analyzing distant evolutionary relationships of very large protein families requires automatic methods for collection of homologous families if one does not wish to transform each new problem case into an art for ingeniously finding the critical links. The FAMILYSEARCHER within the ANNIE environment is the solution for this problem. Since automatic family collection is not corrected on the fly by the watchful eye of an experienced human sequence analyzer, special precautions with regard to removing compositional and repetitive pattern bias, to reciprocal searches and to checking whether hits fall into the same region of starting sequences are necessary to prevent the procedure walking astray. Our experience has shown that the application of the search constraints described in the Methods section has always led to convergence except for the case of known very large groups such as the pancreatic lipase/ATGL/patatin group described in this article (rather a problem of the technical equipment than a principal issue).

In this work, we have identified an ancestral core module consisting of 50–70 residues with a three-strand parallel β-sheet, an α-helix and a turn involving the catalytic serine as substructure with likely common evolutionary origin within the joint classical-lipase/ATGL/patatin cluster. The mere similarity of relative spatial location of some secondary structural elements close to the catalytic serine did not escape the attention of Rydel *et al*. [13], who compared the structures 3TGL (*Rhizomucor miehei *lipase) and 1OXW (patatin) visually and aligned 34 backbone C_α_-atoms. We find that the similarity between the various proteins is limited to the ancestral module (as a result of divergent evolution) but remnant sequence similarity is still detectable with significance. Reduction of overall sequence and structure similarity to a small core module has already been described for other protein families. For example, the Tudor domain "Royal Family" contains a β-β-β-α-3_10 _core with suggested methyl substrate binding function as a common feature of the superfamily [32]. ATGL and classic lipases have fundamental differences with respect to the catalytic mechanism as well as overall fold but both catalyze triglyceride hydrolysis. This suggests that they might have acquired the same function from different predecessor enzymes that are, in turn, derived from the common ancestral module.

If the enzymatic core module is really ancestral, there should be a correspondence with closed loop prototypes that have been described by Berezovsky *et al*. [33-35]. Indeed, prototype P1 and the related prototype P3 have structural (β-α element) and sequence similarity (~30% sequence identity to exemplary sequences) to the ~30 residue region (β_-1_α_+1_) that also encompasses the nucleophilic elbow in the ancestral module (data not shown). In prokaryotes, the P1-containing region corresponds to the P-loop or ATP/GTP-binding motif with the consensus [AG]-x(4)-G-K-[ST]. This tiny-residue motif is similar to the GXSXG stretch followed by further small residues between β_-1 _and α_+1 _in the collected family (Figure 2). The relationship of flanking beta-strands (β_-2 _and β_+1_) with currently described prototypes is unclear. It is possible that they are parts of alternative closed loop structures that might be different between the patatin-like proteins and classic lipases and, thus, exemplifies emergence of/divergence to distinct functions from the common structural ancestor via sequence modification.

Apparently, this β-β-α-β core module was present as an ancestral enzyme that provided basic capabilities for nucleophilic attack mechanisms. In fact, this single mechanism has been evolutionarily extremely successful for many substrates and reaction variants, since the classical-lipase/ATGL/patatin cluster contains numerous enzymes that are neither lipases nor esterases, such as polyketide synthases, dienelactone hydrolases or aminopeptidases (see legend of figure 2 and data not shown).

A striking feature of this mechanism is the discrepancy between high sequence variability and very constrained spatial restrictions for the catalytic center. For proteins that use catalytic triads, the regions that encompass the typical catalytic aspartate and histidine residues are not conserved at all. Not only can the 2D structures of these protein stretches be completely different, but also the sequence positions of these residues relative to the nucleophilic residue (the typical serine) vary considerably. For example, while the catalytic Asp of pancreatic lipase (1N8S, [27]) was shown to reside directly at the C-terminal end of the β_+1 _strand, it is located in long loop region more than 80 residues downstream of the active-site serine in the *C. antarctica *lipase (1TCB, [24]) Nonetheless, the distances of the catalytic residues in the 3D structures of the obtained sequences are relatively constant (for the respective structures in Table 1: SerO-HisN 2.6–3.6Å, HisN-AspCγ 3.3–3.6Å, SerO-AspCγ 7.0–8.4Å), most probably a result of the fact that the enzymatic mechanism crucially depends on a correct spatial arrangement of the catalytic residues.

If proteins carrying these modules indeed shared a common origin, then the sequence variability in the additional regions (that encompass the catalytic amino acids other than the nucleophilic residue) would appear to have enabled the emergence of at least two different enzymatic mechanisms: One relies on a catalytic triad and the other one on a dyad. But which mechanism is the ancestral one? Did the histidine get deleted from the triad, resulting in a protein family that uses Ser-Asp dyads, or was it inserted into an ancestral dyad, leading to the archetypical Ser-His-Asp triad? Assuming an evolution from more simple to more complex mechanisms, the His-insertion version appears more reasonable. The emergence of a catalytically active fold should be more probable if the number of residues that need to be brought into vicinity is limited to two, not three. Considering the evolutionary flexibility regarding the relative positions of the catalytic amino acids in the protein sequences, a third residue may then easily have been inserted during the sequence evolution history. Moreover, not only the regions around the Asp or Asp/His catalytic residues are highly diverse. Various additional modules have been inserted into the sequence, apparently, depending on the physiological environment, substrate specificity or regulation requirements. The ancestral fold template for breaking bonds using nucleophilic attacks seems to have been so "popular" in evolution that it became the origin for an extremely diverse and ubiquitous superfamily of proteins.

## Methods

### FAMILYSEARCHER

We used a multi-step iterative approach to collect a family of related proteins. First, a seed sequence (for example, pancreatic lipase or patatin) is masked by running SEG [36] (parametrization: window length 12 and complexity thresholds K1 = 2.2 and K2 = 2.5) and an own implementation of the COILS algorithm [37] (window length 21, probability threshold 0.5 both for the standard and polar weighting modes) in order to prevent low-complexity and coiled-coil regions from producing evolutionarily unrelated hits. The PSI-BLAST algorithm [4] is then run against the non redundant (nr) database from NCBI to collect an initial family of proteins (matrix BLOSUM62, inclusion cutoff E = 0.001, maximal number of rounds is 10, the internal filter is switched off). The alignment portion of each of the hit sequences is cut out. After adding up to 5 residues of the hit sequence on each side, it is subjected to the same masking procedure, and then fed into the PSI-BLAST algorithm. The previous steps are then repeated in order to gather more distant homologues. With a slight loss of sensitivity but with a dramatic gain in computation speed, it is possible to exclude new database searches with sequences that are highly similar (e.g., 99% sequence identity) to previous start sequences but this shortcut option was not used in this work.

The described procedure carries the risk of picking up unrelated sequences and, consequently, expanding into a large part of the sequence universe. Therefore, we used additional constraints in deciding, which sequences are eligible for family membership and for becoming seeds in the next round. We require a minimum alignment length with the starting sequence of 40 residues and an overlap of the starting segment of at least two thirds. The minimum alignment length and overlap criteria are justified if we assume that the starting sequence represents an individual globular domain, a structural and functional unit. Additionally, we have implemented bi-directionality criteria that we call 'grand-daddy-check' and 'auntie-check'. When a sequence "A" belongs to a set of starting sequences at stage "n" of the family searcher and finds some new hit sequence "B", this "B" enters the set of starting sequences at stage "n+1" of FAMILYSEARCHER. If "B" finds "A" back in its PSI-BLAST searched and the E-value is below a critical value (here: E = 0.01), "B" is called to have passed the grand-daddy-check. If "B" finds not "A" but any other sequence out of the starting set at stage "n" with the critical E-value, it is called to have passed the auntie-check. Obviously, the auntie-check is a more relaxed condition than the grand-daddy check. Our proposed strategy is to first start with a very stringent criterion and to see if the family converges within a certain number of rounds. If this is the case, the more relaxed auntie-check might yield additional members.

In this work, only hits originating from starting sequences that have passed the grand-daddy-check are used for enlarging the cluster at higher stages of FAMILYSEARCHER. This leads to a significant reduction in fanning out. As an example, round 2 identifies 1322 potential sequences for further enlargement but, after applying the grand-daddy-check, only 395 are propagated to the next round. It should be noted that, in our experience of applying the grand-daddy check, most of the protein families converge within a few rounds of the FAMILYSEARCHER. To make this reciprocal checking work at the beginning stage of the procedure, we either generate a new non-redundant database with the user-defined starting sequences included or consider any sequence found with E-value<1.e-8 by the starting segment as "grand daddy". Finally, any family search is limited in the number of rounds to avoid the principally not excluded case of unlimited, excessive expansion of the family by the underlying search algorithm (here: PSI-BLAST).

The FAMILYSEARCHER is one of the integrated algorithms within the ANNIE environment [15] and is available to power users by default. The ANNIE software suite including the FAMILYSEARCHER runs on two 4-processor Opteron SUN VZ40 with 32 GB RAM (an application server and a database server). The sequence-analytic programs (PSI-BLASTs and sequence filters) were computed on a 70 CPU "Opteron" cluster. Both raw results and the ANNIE database were located on a Netapp filer. In order to cope with the large concurrent data streams generated by cluster nodes and the database server, it was inevitable to tune the NFS file system parameters leading to an I/O throughput performance gain of ~30%. Interested partners can apply for assistance in setting up local versions of ANNIE.

### Alignment and phylogenetic tree generation

The selection of appropriate sequences for the multiple alignment of Figure 2 started with a grouping of the set of protein segments collected by FAMILYSEARCHER using the MCL graph clustering algorithm (parameters: inflation 1.002, scheme 7; [38,39]). This procedure, which was performed after the iterative search was completed, allowed us to select a set of representative protein sequences from individual clusters that cover most of the implicated sequence space. The next step consisted in the automatic generation of two separate multiple alignments using the PROBCONS program [40]: one for the selected sequences from the set which was obtained using pancreatic lipase as seed, and the other one for the patatin/ATGL-related sequences. The multiple alignments that were obtained in this way served as an initial guideline but were partially inaccurate in the context of 3D structural considerations and, thus, had to be manually curated. To this end, we generated pairwise structural superpositions between the sequences of each alignment with known crystal structures (see figure 2 for the utilized sequences) and the structure of either pancreatic lipase or patatin as reference. These superpositions were performed using the "fit-selected-residues" functionality of the SWISSPDB-Viewer program [41]. We used the backbone C-atoms of the catalytic nucleophilic residue (serine of cysteine) together with those from the 10 flanking residues as templates. Superposed amino acids from the conserved structural elements were then manually corrected in the multiple alignments. Sequences without resolved 3D structures were aligned to the most closely related protein in the alignment for which the crystal structure was known also with the consideration of pairwise alignments generated by the PSI-BLAST searches. Finally, the alignments were merged into a single alignment and curated using the same procedure.

The phylogenetic tree of the alignment sequences was constructed with the PHYLO_WIN tool [42] using the neighbour joining method [43] in combination with the "observed divergence" distance option from the tool. The 48 positions of the multiple alignment that did not contain any gaps served as a basis for tree calculation. Tree drawing and labelling was done with the TreeGraph program [44].

### 3D structure representation and comparisons

3D protein structures were obtained from the RSCB Protein Data Bank [45]. Analysis and display of these structures was performed using the SWISSPDB-Viewer [41] program. The CE program [21] was used to automatically construct a set of structural superpositions. CE produces Z-scores on the basis of an underlying probability model. These Z-scores are used to estimate the degree of similarity between superposed structures and can be interpreted using significance tables that are provided with each distribution of the CE tool (Z > 4.5: family level similarity; 4.0 – 4.5: superfamily level similarities; 3.7 – 4.0: twilight zone; Z < 3.7: similarities with low significance).

## Abbreviations

ATGL adipose triglyceride lipase, TB terabyte

## Figures and Tables

**Table 1:** Closest hits to known structures from the members of the path in Figure 1. This table lists the known atomic level 3D structures (PDB entry in column 2) that are most closely related to the proteins (accessions in column 1) contained in the path of Figure 1 (found with a PSI-BLAST started with the respective subsequence identified in the FAMILYSEARCHER procedure – round and inclusion E-value in columns 3 and 4). In the last two columns, we present the SCOP and CATH classifiers if available ("-": classifier not found).

accession	PDB entry	round	E-Value	SCOP	CATH
P16233	1GPL	1	0.0	c.69.1.19	3.40.50.1820.28.2.1
AAT95008	1GPL	1	1E-013	-	-
	1ETH	1	6E-11	c.69.1.19	3.40.50.1820.28.6.1
NP_946830	1VJ5	3	2E-12	c.69.1.11	-
	1VA4	3	8E-11	c.69.1.12	-
	1EK1	3	1E-10	c.69.1.11	-
	1A88	3	2E-08	c.69.1.12	3.40.50.1820.12.5.1
	1B6G	3	2E-07	c.69.1.8	3.40.50.1820.6.1.1
NP_771800	1VJ5	3	4E-16	c.69.1.11	-
	1EK1	3	2E-15	c.69.1.11	-
	1UKB	3	4E-14	c.69.1.10	3.40.50.1820.20.1.1
	1VA4	3	1E-13	c.69.1.12	-
	1C4X	3	8E-11	c.69.1.10	3.40.50.1820.50.1.1
YP_049079	1VA4	2	9E-06	c.69.1.12	-
	1JFR	2	2E-04	c.69.1.16	3.40.50.1820.37.1.1
	1TQH	3	2E-17	c.69.1.29	-
	1R1D	3	4E-16	c.69.1.29	-
	1VE7	3	9E-15	-	-
NP_693664	1VA4	2	7E-05	c.69.1.12	-
	1PFQ	3	3E-17	c.69.1.24	3.40.50.1820.32.2.1
	1J2E	3	3E-17	c.69.1.24	3.40.50.1820.32.2.1
	1TKR	3	3E-17	c.69.1.24	3.40.50.1820.32.2.1
	1R9M	3	3E-17	c.69.1.24	3.40.50.1820.32.2.1
NP_296855	1VJ5	5	3E-07	c.69.1.11	-
	1EK1	5	5E-07	c.69.1.11	-
	1A8S	7	4E-07	c.69.1.12	3.40.50.1820.12.4.1
	1VA4	7	7E-07	c.69.1.12	-
	1BRT	7	2E-06	c.69.1.12	3.40.50.1820.12.1.1
NP_799564	1VJ5	3	1E-09	c.69.1.11	-
	1EK1	3	1E-08	c.69.1.11	-
	1A8S	3	5E-08	c.69.1.12	3.40.50.1820.12.4.1
	1VA4	3	6E-08	c.69.1.12	-
	1A8Q	3	3E-07	c.69.1.12	3.40.50.1820.12.3.1
YP_013380	1EHY	2	3E-19	c.69.1.11	3.40.50.1820.42.1.1
	1VA4	2	5E-11	c.69.1.12	-
	1A8S	2	3E-10	c.69.1.12	3.40.50.1820.12.4.1
	1BN6	2	3E-09	c.69.1.8	3.40.50.1820.2.2.1
	1CQW	2	4E-09	c.69.1.8	3.40.50.1820.2.2.2
EAL03661	1OXW	5	1E-11	c.19.1.3	-
CAA27571	1OXW	1	6E-90	c.19.1.3	-

## References

[B1] Zimmermann R, Strauss JG, Haemmerle G, Schoiswohl G, Birner-Gruenberger R, Riederer M, Lass A, Neuberger G, Eisenhaber F, Hermetter A, Zechner R (2004). Fat mobilization in adipose tissue is promoted by adipose triglyceride lipase. Science.

[B2] Birner-Gruenberger R, Susani-Etzerodt H, Waldhuber M, Riesenhuber G, Schmidinger H, Rechberger G, Kollroser M, Strauss JG, Lass A, Zimmermann R, Haemmerle G, Zechner R, Hermetter A (2005). The lipolytic proteome of mouse adipose tissue. Mol Cell Proteomics.

[B3] Zechner R, Strauss JG, Haemmerle G, Lass A, Zimmermann R (2005). Lipolysis: pathway under construction. Curr Opin Lipidol.

[B4] Altschul SF, Madden TL, Schaffer AA, Zhang J, Zhang Z, Miller W, Lipman DJ (1997). Gapped BLAST and PSI-BLAST: a new generation of protein database search programs. Nucleic Acids Res.

[B5] Anthonsen HW, Baptista A, Drablos F, Martel P, Petersen SB, Sebastiao M, Vaz L (1995). Lipases and esterases: a review of their sequences, structure and evolution. Biotechnol Annu Rev.

[B6] Lowe ME (2002). The triglyceride lipases of the pancreas. J Lipid Res.

[B7] Haemmerle G, Zimmermann R, Zechner R (2003). Letting lipids go: hormone-sensitive lipase. Curr Opin Lipidol.

[B8] Mead JR, Irvine SA, Ramji DP (2002). Lipoprotein lipase: structure, function, regulation, and role in disease. J Mol Med.

[B9] Holmquist M (2000). Alpha/Beta-hydrolase fold enzymes: structures, functions and mechanisms. Curr Protein Pept Sci.

[B10] Ollis DL, Cheah E, Cygler M, Dijkstra B, Frolow F, Franken SM, Harel M, Remington SJ, Silman I, Schrag J, . (1992). The alpha/beta hydrolase fold. Protein Eng.

[B11] Ganal MW, Bonierbale MW, Roeder MS, Park WD, Tanksley SD (1991). Genetic and physical mapping of the patatin genes in potato and tomato. Mol Gen Genet.

[B12] Vancanneyt G, Sonnewald U, Hofgen R, Willmitzer L (1989). Expression of a Patatin-like Protein in the Anthers of Potato and Sweet Pepper Flowers. Plant Cell.

[B13] Rydel TJ, Williams JM, Krieger E, Moshiri F, Stallings WC, Brown SM, Pershing JC, Purcell JP, Alibhai MF (2003). The crystal structure, mutagenesis, and activity studies reveal that patatin is a lipid acyl hydrolase with a Ser-Asp catalytic dyad. Biochemistry.

[B14] Dessen A, Tang J, Schmidt H, Stahl M, Clark JD, Seehra J, Somers WS (1999). Crystal structure of human cytosolic phospholipase A2 reveals a novel topology and catalytic mechanism. Cell.

[B15] Schneider G, Wildpaner M, Kozlovszky M, Kubina W, Leitner F, Novatchkova M, Schleiffer A, Sun T, Eisenhaber F (2005). The ANNOTATOR software suite. http://www.iscb.org/ismb2005/demos/15.pdf.

[B16] Bork P, Dandekar T, Diaz-Lazcoz Y, Eisenhaber F, Huynen M, Yuan Y (1998). Predicting function: from genes to genomes and back. J Mol Biol.

[B17] Schleiffer A, Kaitna S, Maurer-Stroh S, Glotzer M, Nasmyth K, Eisenhaber F (2003). Kleisins: a superfamily of bacterial and eukaryotic SMC protein partners. Mol Cell.

[B18] Eisenhaber F, Wechselberger C, Kreil G (2001). The Brix domain protein family -- a key to the ribosomal biogenesis pathway?. Trends Biochem Sci.

[B19] Novatchkova M, Leibbrandt A, Werzowa J, Neubuser A, Eisenhaber F (2003). The STIR-domain superfamily in signal transduction, development and immunity. Trends Biochem Sci.

[B20] Novatchkova M, Eisenhaber F (2004). Linking transcriptional mediators via the GACKIX domain super family. Curr Biol.

[B21] Shindyalov IN, Bourne PE (1998). Protein structure alignment by incremental combinatorial extension (CE) of the optimal path. Protein Eng.

[B22] Derewenda U, Brzozowski AM, Lawson DM, Derewenda ZS (1992). Catalysis at the interface: the anatomy of a conformational change in a triglyceride lipase. Biochemistry.

[B23] Wei Y, Contreras JA, Sheffield P, Osterlund T, Derewenda U, Kneusel RE, Matern U, Holm C, Derewenda ZS (1999). Crystal structure of brefeldin A esterase, a bacterial homolog of the mammalian hormone-sensitive lipase. Nat Struct Biol.

[B24] Uppenberg J, Hansen MT, Patkar S, Jones TA (1994). The sequence, crystal structure determination and refinement of two crystal forms of lipase B from Candida antarctica. Structure.

[B25] Nardini M, Lang DA, Liebeton K, Jaeger KE, Dijkstra BW (2000). Crystal structure of pseudomonas aeruginosa lipase in the open conformation. The prototype for family I.1 of bacterial lipases. J Biol Chem.

[B26] Jeong ST, Kim HK, Kim SJ, Chi SW, Pan JG, Oh TK, Ryu SE (2002). Novel zinc-binding center and a temperature switch in the Bacillus stearothermophilus L1 lipase. J Biol Chem.

[B27] van Tilbeurgh H, Egloff MP, Martinez C, Rugani N, Verger R, Cambillau C (1993). Interfacial activation of the lipase-procolipase complex by mixed micelles revealed by X-ray crystallography. Nature.

[B28] Pathak D, Ollis D (1990). Refined structure of dienelactone hydrolase at 1.8 A. J Mol Biol.

[B29] Kim KK, Song HK, Shin DH, Hwang KY, Choe S, Yoo OJ, Suh SW (1997). Crystal structure of carboxylesterase from Pseudomonas fluorescens, an alpha/beta hydrolase with broad substrate specificity. Structure.

[B30] Devedjiev Y, Dauter Z, Kuznetsov SR, Jones TL, Derewenda ZS (2000). Crystal structure of the human acyl protein thioesterase I from a single X-ray data set to 1.5 A. Structure Fold Des.

[B31] Sanishvili R, Yakunin AF, Laskowski RA, Skarina T, Evdokimova E, Doherty-Kirby A, Lajoie GA, Thornton JM, Arrowsmith CH, Savchenko A, Joachimiak A, Edwards AM (2003). Integrating structure, bioinformatics, and enzymology to discover function: BioH, a new carboxylesterase from Escherichia coli. J Biol Chem.

[B32] Maurer-Stroh S, Dickens NJ, Hughes-Davies L, Kouzarides T, Eisenhaber F, Ponting CP (2003). The Tudor domain 'Royal Family': Tudor, plant Agenet, Chromo, PWWP and MBT domains. Trends Biochem Sci.

[B33] Berezovsky IN, Trifonov EN (2001). Van der Waals locks: loop-n-lock structure of globular proteins. J Mol Biol.

[B34] Berezovsky IN, Kirzhner A, Kirzhner VM, Trifonov EN (2003). Spelling protein structure. J Biomol Struct Dyn.

[B35] Berezovsky IN, Kirzhner A, Kirzhner VM, Rosenfeld VR, Trifonov EN (2003). Protein sequences yield a proteomic code. J Biomol Struct Dyn.

[B36] Wootton JC (1994). Non-globular domains in protein sequences: automated segmentation using complexity measures. Comput Chem.

[B37] Lupas AN, Gruber M (2005). The structure of alpha-helical coiled coils. Adv Protein Chem.

[B38] Enright AJ, Van Dongen S, Ouzounis CA (2002). An efficient algorithm for large-scale detection of protein families. Nucleic Acids Res.

[B39] Dongen S (2005). Graph Clustering by Flow Simulation.

[B40] Do CB, Mahabhashyam MS, Brudno M, Batzoglou S (2005). ProbCons: Probabilistic consistency-based multiple sequence alignment. Genome Res.

[B41] Guex N, Peitsch MC (1997). SWISS-MODEL and the Swiss-PdbViewer: an environment for comparative protein modeling. Electrophoresis.

[B42] Galtier N, Gouy M, Gautier C (1996). SEAVIEW and PHYLO_WIN: two graphic tools for sequence alignment and molecular phylogeny. Comput Appl Biosci.

[B43] Saitou N, Nei M (1987). The neighbor-joining method: a new method for reconstructing phylogenetic trees. Mol Biol Evol.

[B44] Muller J, Muller K (2004). TreeGraph: automated drawing of complex tree figures using an extensible tree description format. Molecular Ecology Notes.

[B45] Berman HM, Westbrook J, Feng Z, Gilliland G, Bhat TN, Weissig H, Shindyalov IN, Bourne PE (2000). The Protein Data Bank. Nucleic Acids Res.

